# Designing and Validating a Questionnaire on Healthy Lifestyle to Reduce Depressive Symptoms among Adolescents

**Published:** 2020-01

**Authors:** Esra Tajik, Latiffah Abd Latiff, Chin Yit Siew, Hamidin Awang, Siti Nur' Asyura Adznam

**Affiliations:** 1 Department of Community Nutrition, National Nutrition and Food Technology Research Institute, School of Nutrition Sciences and Food Technology, Shahid Beheshti University of Medical Sciences, Tehran, Iran.; 2 Psychiatry and Psychology Research Center, Tehran University of Medical Sciences, Tehran, Iran.; 3 Department of Nutrition and Dietetics, School of Medicine and Health Sciences, University Putra Malaysia, Serdang, 43400, Selangor, Malaysia.; 4 Department of Community Health, School of Medicine and Health Sciences, Universiti Putra Malaysia, Serdang, 43400, Selangor, Malaysia.; 5 Department of Psychiatry, School of Medicine and Health Sciences, Universiti Putra Malaysia, Serdang, 43400, Selangor, Malaysia.

**Keywords:** *Depressive Symptom*, *Lifestyle*, *Partial Least Squares*, *Reliability*, *Validity*

## Abstract

**Objective:** Most adolescents do not meet national recommendations for healthy lifestyle and reducing depressive symptom. A comprehensive educational program with its specified questionnaire is needed to improve healthy lifestyle to assess the lifestyle components. This study aimed to confirm the validity of a healthy lifestyle questionnaire to assess depressive symptoms among adolescents.

**Method** **:** A descriptive predictive study using simple random sampling was performed in a secondary school in Kuala Lumpur, Malaysia, among 235 school-going adolescents (13-15 years old) with Malaysian nationality. The instrument consisted of a set of dual language (English and Malay) for both researcher-administered questionnaires (knowledge, attitude, eating behavior, and relaxation activities) and standard-validated questionnaires (Physical Activity questionnaire for Adolescents, Depression, Anxiety, and Stress Scale- 21, and Brief Copping). Data were analyzed using the kappa statistics (k) and the intraclass correlation coefficient test for reliability and Partial Least Squares (PLS) for validity.

**Results: **The reliability of all variables was over the substantial level (ICC and/or k > 0.61). The model and construct variables were predictive of depressive symptoms by 49.4%. To analyze the validity of the questions, 11 questions were removed from the initial model for factorial loading less than 0.5. In convergent validity of theory of information-motivation-behavioral skills, AVE (average variance construct), the outer loading, was higher than 0.5.

**Conclusion: **PLS confirmed the existence of sufficient correlations between different items of the construct. Thus, the weights of indicators appeared to be appropriate indicators for the model. The results proved that the information, motivation, and behavioral skills model was efficient for healthy lifestyle and can be a good base for further research.

Although psychosocial problems and unhealthy lifestyles are major problems among adolescents, they are often neglected (1, 2). Lifestyle education is one of the solutions; however, most adolescents do not receive proper recommendations (3). 

A comprehensive educational program requires a suitable questionnaire set, based on a specified theory, to assess the lifestyle components to reduce mental disorders (4-6). Moreover, it should have minimal unrelated variables and focus on the important variables to obtain the appropriate correlations (7). 

The need to assess the main components of lifestyle that predict items for depressive disorders through standards in adolescents, lead to the development of questionnaires (8, 9). Thus, researchers gathered a combination of true or false and/or healthy or unhealthy of knowledge, attitude and behaviors in terms of lifestyle in adolescents (10-12). Many factors influence adolescents’ lifestyle. Understanding these influences helps to develop the questionnaires and the educational module. Other lifestyle educational studies lack comprehensive recommendations such as not missing breakfast, eating more antioxidants and anti-stress minerals/vitamins, working out at least 3 times a week, and learning stress management skills (10, 11). 

Previous studies have shown that a healthy lifestyle is associated with mental health (4, 13). In addition, some other studies indicated that a healthy lifestyle intervention consists of healthy eating, physical activity, and having stress management skills (5, 6). Eating behavior, dietary intake, physical activity and exercise have positive effects on treatment and prevent stress (7-9, 14). Previous studies have found that the theory of information-motivation-behavioural skills (IMB) is an appropriate model to conduct a study and collect data on adolescents (15-17). By using efficient interventions, enough information and knowledge, motivation and providing choices, attitude can be improved and behavioral skills will develop (15, 18). The IMB model has been tested for healthy lifestyle behaviors to predict physical activity and dietary intake in adolescents (12). 

Planning and evaluating effectiveness of an incisive healthy lifestyle intervention on adolescents requires identification of its related factors and needs assessment. Based on our knowledge, there are no validated tools to identify the knowledge and attitude of adolescents on 3 main components of lifestyle: a healthy diet, physical activity, and stress management skills (12, 14). 

There has been a lack of validated and reliable questionnaire to assess the knowledge, attitude, and practice on lifestyle factors for a healthy lifestyle intervention to reduce stress symptoms among adolescents based on the theory of IMB. Thus, the aim of this study was to conduct the validity of the Healthy Lifestyle (healthy eating, physical activity, and stress management skills) Questionnaire to assess depressive symptoms among adolescents using the Partial Least Squares (PLS). 

***Conceptual Model***

The present study used IMB theory to develop a module, including knowledge, attitude, and behavioral factors (eating, physical activity, relaxation activities, and coping) (15, 17). The theory of IMB is an appropriate model with fewer factors to conduct a study and collect data on adolescents. With efficient intervention using enough information and knowledge, motivation, and choices, attitude can be improved and behavioral skills will develop (15, 18). The IMB model implies that people who are well-informed, motivated to act and receive behavioral skills will maintain health-promoting behaviors and experience proper health outcomes (16). Based on the model, if information and knowledge is understandable, it will lead to successful health behavior. Adequately informed and motivated adolescents will learn the necessary skills to develop health promoting behaviors. The relationships in the IMB model were confirmed with all structural paths being significant. Baronowski et al (15) reviewed psychosocial correlates of dietary intake noting that models with knowledge and attitude can predict dietary intake (15). The theoretical framework of the present study according to the information-motivation-behavioral skill model is shown in Figure 1.

## Materials and Methods

A predictive study was conducted on school-going adolescents at a secondary school “SMK Aminuddin Baki” in Kuala Lumpur, Malaysia, in September 2014. Out of 5 levels at the secondary school, students at levels 1, 2, and 3 were selected using simple random sampling. The inclusion criteria included age 13-15 years, and Malaysian nationality from all ethnic groups. A total of 235 students met the inclusion criteria and informed consent was obtained from all before their participation. 

A set of dual-language (English and Malay) researcher-administered questionnaires were developed to obtain comprehensive information on demographic variables, such as age, gender, ethnicity, religion, and knowledge and attitude about healthy lifestyle, healthy diet, physical activity, stress management skills, relaxation activities, and eating behaviors. Furthermore, some of the questionnaires used in this study were standard and validated and set to cover the practice part of the questionnaire, including PAQ-A (Physical Activity Questionnaire for Adolescents), Brief Coping (to measure 14 coping strategies) and DASS-21 (to measure symptoms of depression, anxiety, and stress). 

***Questionnaires***

Three domains of IMB model, including information or knowledge, motivation and attitude, and behavior or practice were introduced into 3 main components of lifestyle: a healthy diet, physical activity, and stress management skills (coping and relaxation activities). Each parameter (healthy diet, physical activity, and stress management skills) needed all the 3 domains (knowledge, attitude, and practice). 

For questionnaires on knowledge, attitude, and practice, some items were taken from existing questionnaires and some from the extensive review of literature to ensure that the questionnaire set was representative of all domains being measured. However, for questionnaires on practice, some were standard and validated, including PAQ-A, and Brief Coping. Moreover, as the last parameter of the theory and the main goal of the study, reducing stress symptoms was evaluated using DASS-21. The permission to use questionnaires was obtained through email from the original founder of each questionnaire.

A pool of 120 items and the process of translation to and from Malay were generated by a group of nutritionists. A review in both English and Malay was performed by a panel of 6 experts in community health, nutrition, psychiatry, and psychology. This process reduced the number of questions to 99 items. Questionnaire sets were discussed individually with 25 students aged 13-15 years to clarity the meaning of the questionnaire. Comments were sought related to understandability of the questions and ease of filling out the questionnaire. Then, standard questionnaires (PAQ-A (9 items), Brief Coping (28 items), and DASS-21 (21 items)) were added to the questionnaire set. The total number of the questions reached 157.

The final questionnaire set was self-administered in 10 main sections (A to E) (Table 1). A total of 210 students were invited to complete the questionnaire set. Section A as a first section contained sociodemographic factors, including age, ethnicity, religion, family members, educational level, parents’ occupations, household income, and type of house. The other sections contained researcher-administered questionnaires (99 items before validation and 85 items after validation, B1-D2) and standard questionnaires (58 items, D3-E) related to various topics as shown below:

Section B1- Knowledge on lifestyle, diet, and physical activity (knowledge on LS, D and PA): The questionnaire was developed and modified based on literature review (20-23). It includes 22 questions which aimed to assess the knowledge on healthy lifestyle (3 items), nutrition and healthy diet (16 items), and physical activity (3 items). This questionnaire has 5 multiple-choice questions with only 1 true answer. 

Section B2- Knowledge on stress management skills (knowledge on SM): The questionnaire was developed based on literature review (24-26). It has 15 questions which assess the knowledge on stress management skills (self-identification (3 items), relaxation activity (3 items), coping stress (6 items), and moral values (3 items)). This questionnaire has 5 multiple-choice questions with only 1 true answer. 

Section C1- Attitude on lifestyle, nutrition, diet, and physical activity (Attitude on LS, D and PA): This questionnaire with 17 questions was developed to assess the adolescents’ attitude toward lifestyle (3 items), diet (11 items), and physical activity (3 items). The items were based on previous studies’ questionnaires (20-21, 23, 27, 28). The 5-point scale scores were used and the scores ranged from ‘strongly agree (1 point)’ to ‘strongly disagree (5 points)’. The questions were in negative worded items, except the fifth and sixth questions (eating behavior). The reverse items (questions 5 and 6) were considered for summing the response values. 

Section C2- Attitude on stress management skills (Attitude on SM): This questionnaire contains 15 questions according to previous studies and literatures (24, 25, 29-34). Questions measure self-identification (3 items), relaxation activity (3 items), coping stress (6items), and moral values (3 items). All the items have 5 response choices, ranging from ‘strongly agree (1 point)’ to ‘strongly disagree (5 points)’. 

Section D1- Eating behavior (EB): Meal skipping and snacking behaviors were assessed using a self-administered questionnaire. The questions were developed and modified based on Eating Behaviors Questionnaire (EBQ) (35) and food habits from another study (22). The questionnaire assessed the frequency of meal consumption and snacking between meals, frequency of eating outside, take-away food, fast foods, sweet foods and beverages, and frequency of food groups (fruits, vegetables, dairy, and fish), type of snacks consumption, and use and type of dietary supplement. Adolescents with skipping at least one meal per day were considered as skipping meal and snacking group (36-38). 

Section D2- Relaxation activity (RA): The frequency of practicing relaxation activity techniques for muscle relaxation activity and deep breathing activity based on literature review (39, 40) was assessed using 2 questions: “During the last week, how many times did you do muscle relaxation activity?” and “During the last week, how many times did you do deep breathing activity?” The frequency of relaxation activities was as follows: none, 1 time, 2 or 3 times, 4 or 5 times, and 6 or 7 times. Score 1 was given if they practiced every day and score 0 if they practiced less than 7 times per week. 

Section D3- PAQ-A (PA): Physical activity of respondents was assessed using the modified and adopted Physical Activity Questionnaire for Adolescents (PAQ-A) (41, 42). The instrument consisted of 9 items: leisure time activity during the last 7 days with 5-point scale, ranging from ‘no’ activity to ‘7 times or more’. Items 2 to 7 related to activities of the adolescents, which ranged from 1 (lowest activity response) to 5 (the highest activity response). 

For item 8, the adolescents are asked about the frequency of participating in daily physical activity in the previous week, and it is scored from 1 ‘none’ to 5 ‘very often’. The mean physical activity score for all days of the week are calculated to form a composite score for item 8. Finally, item 9 identifies adolescents with unusual activities during the previous week. Once the sum of the scores from items 1 to 8 are calculated, the final PAQ-A activity summary score can be obtained by considering the mean of these 8 items. All adolescents can be classified into 3 categories: (1) those with low physical activity, with the mean score 1 to 2.33; (2) with moderate physical activity, with the mean score 2.34 to 3.66; and (3) with high physical activity, with the mean score 3.67 to 5, based on their mean total physical activity score (41, 43, 44). At last, those with high and moderate activity scored 1 and those with low activity scored 0.

Section D4- Brief coping: It is a valid and reliable instrument in identifying coping strategies of secondary school adolescent. The original Brief COPE (45) was translated into Malay language (46). The scale is rated by a 4-point Likert scale and includes 28 items, ranging from 1 (I have not been doing this at all) to 4 (I have been doing this a lot). The higher score represents greater coping strategies used by the respondents. In total, 14 dimensions (2 items / dimension) and/or 14 strategies (self-distraction, active coping, denial, substance abuse, use of emotional support, use of instrumental support, behavioral disengagement, venting, positive reinterpretation, planning, humor, acceptance, religion, self-blame) are put forward by this scale. Each strategy is scored from 2 to 8. Reliability analysis was applied to test internal consistency of the Malay Brief COPE (47). The total Cronbach's alpha value of the Malay version Brief COPE was 0.83 and most of the items were loaded nicely according to the coping strategies. 

Section E- Depression, Anxiety, and Stress Scale (DASS-21): This questionnaire measures perception of depression, anxiety, and stress among adolescents. Also, it was validated in Malaysia among adolescents. The internal consistency for stress, anxiety, and depression was 0.68, 0.67, and 0.70, respectively (48). It consists of 21 questions (7 items to evaluate each domain) which are scored based on a 4-point Likert scale, ranging from 0 (Did not apply to me at all) to 3 (Apply to me very much, or most of the time) (49). Once multiplied by 2, each score can be transferred into the DASS profile sheet (Table 2).

***Reliability***

A reliability analysis was run to determine the items to be rejected or retained and to check the internal consistency of the questionnaires. The reliability of the questionnaire was determined using test-retest reliability among 35 secondary school students in SMK Kota Masai (not included in the main data collection). The questionnaire was distributed twice among students with an interval of 14 days between the first and second administration. In the second administration, questions, except for the demographic factors, were repeated for each individual. Internal consistency of the questionnaire was determined using test-retest reliability. The value of kappa was ranged for dummy variables as knowledge on lifestyle, diet, and physical activity (0.65-0.92), knowledge on stress management skills (0.68-0.89), eating behavior (0.72-0.91), physical activity (0.68-0.91), and relaxation activity practice (0.73-0.83). 

The value of ICC for continuous variables is as follows: attitude of lifestyle, diet, and physical activity (0.80-0.97); attitude of stress management skills (0.77-0.95); DASS-21 (0.85-0.97); and Coping strategies (0.78-0.95). The items were significant enough not to be deleted based on reliability test.

***Validity***

***Content Validity***

The validity of questionnaires indicates which questionnaire exactly measures the data according to the goal of the study. To select the best clarity for the questions the followings should be considered: knowledge; attitude and practice for nutrition; physical activity; and psychology parts, which were performed by health professionals and the expert panel. At this step, the clarity of meaning, language, the flow of contents, usefulness of the questions, and the required time to answer were checked. Content validity was determined by 6 health professionals, lecturers, and specialists in nutrition, health, and psychology from the Community Health Department and Nutrition and Dietetic Department of Universiti Putra Malaysia. Based on the recommendations of the expert team, improvements were made, and questions with unclear items were modified. 

All 6 panelists were asked to respond to each item in the questionnaire. The rating included 2 options. One of them was essential and useful and the other was nonessential and unnecessary. To decide which item should be kept, modified and/or deleted, the following formula for the content validity ratio (CVR) is calculated (50):


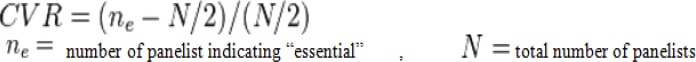


CVR ranged from +1 to -1. Positive value indicates that more than half of the panelists voted “essential”. CVR for questionnaires of the present study was positive, except for 3 questions (2 questions about antioxidants in the knowledge part and the first question in the attitude part), which were corrected and modified. The results of the CVR test are described in Table 3 and indicate that the remaining items have CVR of +0.333 to +1.

***Construct Validity***

The relationship between the factors adopted in the modeling method was verified using Structural Equation Modeling (SEM). The SEM method is a technique used to study complex relations that initiate factors (latent variables) that cannot be directly measured (51, 52). Dependency relations in SEM are used simultaneously as a dependent variable and can turn into an independent variable in subsequent relations of dependency (52). 

The SEM or structural model involves dependent and independent variables, measurement, and structural theories. The theory defines how the variables represent, systematically and logically, the use of constructs in a theoretical model. Thus, the theory determines relationships which show how the variables indicate latent constructs (53, 54). The ellipses are the independent (exogenous) and dependent (endogenous) variables that are latent variables, or the constructs of the study. The rectangles are indicators or variables, and the arrows show the effect of variables; this measurement model identifies the unique relationship between the variables (55). 

The partial least squares (PLS) is one of the major techniques in SEM and has 6 steps: model specification, identification, data collection, estimation, re-specification, and the reporting of the results (56). The specifications of the model represent the hypothesis which in this case is conceptual framework. Identification means that the experimental study should be supported by the theory, and the third stage is the data collection.

The PLS-SEM was run and the results such as convergence, reliability, and validity were reported. The type of reliability coefficient is most similar to the Cronbach’s alpha test which measures internal consistency. Moreover, discriminant validity in PLS-SEM was measured to show which given construct is different from the other. Each construct AVE (Average Variance Extracted) should be larger than its correlation with other constructs, and each item should have a higher load on its assigned construct than on the other constructs (53). Moreover, the Goodness-of-Fit (GOF) index is the key standard for evaluating the inner structural model.

***Data analysis***

Data were analyzed using SPSS 21.0 software and Smart PLS (Partial Least Square – Path Modeling, 2.0.3) software for descriptive analysis and running reliability. Test-retest reliability for dummy variables was determined using kappa statistics (k) (57, 58) and continuous data or questions with Likert scale using intraclass correlation coefficient (ICC). Kappa and ICC were interpreted according to the scale proposed by Landis and Koch (59) including: score <0.20 (poor correlation), fair (0.21-0.40), moderate (0.41-0.60), substantial (0.61-0.80) and near perfect (0.81-1.00). Moreover, for factor loading SPSS and for the rest of the validating process pls were used. The factor loading greater than 0.5 was an accepted value (60). 

## Results

***Descriptive statistics***

A total of 201 valid data with no missing values were obtained out of 235 respondents (response rate of 85.5%). The results indicated a dominant proportion of female (70.7%), Muslim (69.6%), and Malay (30.8%) students. The average age was 13.8 ± 0.42 years.

***Validity***

At first, only the construct of the researcher-administered questionnaires (Table 4) were entered into the analysis. Three questions were excluded as a result of the CVR test. For the rest, SEM analysis was performed with all latent variables with partial estimation in PLS-Path Modeling. The results and graphs of PLS on reflective (knowledge and attitude) and formative (EB and RA) models are shown in Figure 2.

Before entering variables into the PLS model, according to factor loads and weights, some variables may have to be excluded from the model. Factors in the initial model for knowledge and attitude had factor loading between 0.500 to 0.945 except 11 questions which had factor loading less than 0.5, including knowledge of lifestyle (2 variables, 0.341 and 0.320); diet (2 variables, 0.494 and 0.448); physical activity (1 variable, 0.439); stress management (2 variables, 0.398 and 0.417); attitude on lifestyle (1 variable, 0.459); diet (2 variables, 0.471 and 0.403); and stress management (1 variable, 0.383). Hence, all 11 questions were removed and the initial model with 96 indicators was fixed with 85 indicators for the necessity of the adjustment. After excluding the variables, data were analyzed and new results in the structural model were obtained. All variable constructs showed factorial loads higher than 0.7, which indicates that there is convergent validity (Figure 2). In addition, all remaining variables are entered into PLS to check if the model is fit.

***Goodness-of-Fit Index***

Goodness-of-fit (GOF) was applied as an index to verify that the model fitted the empirical data. The GOF values were between 0 to 1, with 0.10 (small), 0.25 (medium), and 0.36 (large). The GOF is the mean value of the average communality (AVE values) and the average R2 value(s). According to Table 5, in this study, the GOF index was measured as 0.551, which shows that empirical data fit the model satisfactorily and has substantial predictive power. 

The standardized root mean square residual (SRMR) is an index to measure and estimate whether the model fits. When SRMR is ≤ 0.08, the study model has a good fit. This study model’s SRMR was 0.06, which revealed that the model had a good fit, whereas the Chi-square was equal to 2817.082 and NFI equal to 0.810.

Constructs, including researcher-administered questionnaires, were entered into the model (Figure 2) to assess the convergent validity. Also, AVE for reflective questionnaire was checked to be higher than 0.5 (greater than 50%), which meets the recommended criterion (61) (Table 5), and for the formative questionnaire, the outer loading should be more than 0.5. Considering the criteria for the defined adjustments, there were no values lower than those recommended.

The next step was to analyze the validity of the theory of the IMB model. All constructs (researcher-administered questionnaires and standard questionnaires) were entered into the model (Figure 3). Next, the initial model was tested according to the conceptual framework, and since it was done after fixing factors and items in the previous level, none of the variables were removed from the model. Thus, the initial model and the fixed model were the same. AVE for the reflective questionnaire and the outer loading for the formative questionnaire should be higher than 0.5. In this model, the criterion recommended was applied and there were no values lower than those recommended. The outer weight and outer loading of factors includes eating behavior (outer weight 0.415, outer load 0.630, SE 0.276, t-value 2.218), physical activity (outer weight 0.631, outer load 0.719, SE 0.171, t-value 3.189), relaxation activity (outer weight 0.375, outer load 0.571, SE 0.175, t-value 2.372).

The discriminant validity determines the adequacy of discrimination of the variable. The test is performed using the square root of the AVE, which must be higher than the correlation between the constructs (62). 

**Table 1:** The Questionnaire Set in Details for Numbering, Parts of Questionnaire Set and Scoring

**Questions**	**Parts**	**Questions**	**Score**
A	Sociodemographic	15	Report response
B 1	Knowledge on LS^a^, D^b^, PA^c^	29	True-false
B 2	Knowledge on SM^d^	17	ʺ
C 1	Attitude on LS, D, PA	21	Summing up the total selected statements
C 2	Attitude on SM	16	ʺ
**Behavior**			
D 1	EB^e^	17	Recode into scores. Dummy variables
D 2	RA^f^	2	ʺ
D 3	PA	9	Summing up the total selected statements and categorizing them into low, moderate, high
D 4	Brief coping	28	Each 2 questions represent one strategy with the score of 2-8.
E	DASS^g^	21	Summing up the total selected statements and categorizing each domain to normal, mild, moderate, severe, and extremely severe

a LS, lifestyle;

b D, diet;

c PA, physical activity;

d SM, stress management

e EB, eating behavior

f RA, relaxation activity

g DASS, depression, anxiety, stress scale

**Table 2:** The Score Ranging for Symptoms of Depression, Anxiety and Stress Based on DASS-21

**Severity**	**Depression**	**Anxiety**	**Stress**
Normal	0-9	0-7	0-14
Mild	10-13	8-9	15-18
Moderate	14-20	10-14	19-25
Severe	21-27	15-19	26-33
Extremely severe	28 +	20 +	34 +

**Table 3:** Calculating CVR for All Questionnaire Parts at the First Round of Judgment

**Questions**	**n**_e_	**CVR**	**Interpretation**
Knowledge 1-7	6	+1	Remained
Knowledge 8, 10-15, 17-22, 25-46	5	+0.66	Remained
Knowledge 9, 16	4	+0.33	Remained
Knowledge 23-24	2	-0.33	Eliminated
Attitude 1	2	-0.33	Eliminated
Attitude 2-11, 14-16, 21-35	6	+ 1	Remained
Attitude 12-13, 17-20, 36-37	4	+0.33	Remained
Eating behavior 1-10	6	+ 1	Remained
Eating behavior 11-14	5	+0.66	Remained
Relaxation activity 1-2	6	+ 1	Remained

**Table 4:** Lifestyle Questionnaire with Its Section Details and Scales

**Construct**	**Indicators**	**Scale**
Knowledge on LS, D, and PA		
1	Healthy lifestyle:	Multiple Choice
2	Healthy lifestyle.	Multiple Choice
3	Which one is NOT TRUE about healthy lifestyle?	Multiple Choice
4	Which one is TRUE about healthy lifestyle?	Multiple Choice
5	Healthy lifestyle can make the body …	Multiple Choice
6	Balanced food intake and physical activity:	Multiple Choice
7	A balanced diet:	Multiple Choice
8	Which one is NOT TRUE about healthy diet?	Multiple Choice
9	You will get all the nutrients needed by?	Multiple Choice
10	According to the food pyramid, the types of foods you should eat at least is?	Multiple Choice
11	Consuming food with excess sugar content:	Multiple Choice
12	Omega-3 can:	Multiple Choice
13	The best source of omega 3 is?	Multiple Choice
14	Which kind of drinks are good for health?	Multiple Choice
15	The followings are the effect of missing meal except:	Multiple Choice
16	With missing breakfast:	Multiple Choice
17	Which one is correct about breakfast?	Multiple Choice
18	Excess energy (calorie) intake can lead to?	Multiple Choice
19	Food that rich in vitamin, mineral and fibre are?	Multiple Choice
20	Which food group can improve mental health?	Multiple Choice
21	Antioxidant is important for?	Multiple Choice
22	Which one is NOT TRUE about antioxidents?	Multiple Choice
23	Which fruits have more antioxidants?	Multiple choice *
24	Which color(s) shows more antioxidants?	Multiple choice *
25	The main source of antioxidant is?	Multiple Choice
26	Which kind of activity is advised to do every day as many as we can?	Multiple Choice
27	Which kind of activity is not advised to do more than one hour per day?	Multiple Choice
28	Which one is NOT the benefit of physical activity on health?	Multiple Choice
29	Which activity can improve our body health?	Multiple Choice
Knowledge on SM		
30	Identifying ourselves is?	Multiple Choice
31	Identifying weakness or strength of ourselves is?	Multiple Choice
32	Identifying ourselves can lead to?	Multiple Choice
33	Relaxation activity is?	Multiple Choice
34	Muscle relaxation activity is?	Multiple Choice
35	With doing relaxation activity, I:	Multiple Choice
36	Which one is NOT TRUE about stress?	Multiple Choice
37	Which one is TRUE about stress?	Multiple Choice
38	Which one is NOT the normal reaction to stress?	Multiple Choice
39	Select the TRUE statement.	Multiple Choice
40	Which one is NOT TRUE?	Multiple Choice
41	Learning stress management is not necessary because …	Multiple Choice
42	Which one is NOT a strategy for stress management?	Multiple Choice
43	Stress management components are …	Multiple Choice
44	Which answer has moral values?	Multiple Choice
45	What will happen if we DON’T practice moral values in our life?	Multiple Choice
46	Which one is NOT TRUE about moral values practices?	Multiple Choice
Attitude on LS, D, and PA		
1	Healthy lifestyle can change my life as well as my health.	Likert 1-5*
2	I have accepted the lifestyle of my parents, so no need to change it.	Likert 1-5
3	I am pleased to change my lifestyle as I’ve seen its consequences.	Likert 1-5
4	I am healthy so I don’t need to eat healthy.	Likert 1-5
5	Healthy lifestyle is no meaningful for me.	Likert 1-5
6	healthy eating is difficult and hard to do	Likert 1-5
7	Healthy diet was useful for our ancestors.	Likert 1-5
8	I have to prevent eating food and drinks with high sugar.	Likert 1-5
9	I feel guilty after eating high sugar and fried foods.	Likert 1-5
10	The balanced diet cannot improve our health.	Likert 1-5
11	Fast food is a must have for me.	Likert 1-5
12	Skipping a meal is not important to me.	Likert 1-5
13	Breakfast is very important; it helps me to eat more at lunch time.	Likert 1-5
14	I do not care about the drinks. All kind of drinks are good for health.	Likert 1-5
15	Sweets can make me happier, so I eat them a lot.	Likert 1-5
16	I don’t need fruits and vegetables; I can get nutrients from other foods.	Likert 1-5
17	Vegetables are not important for me.	Likert 1-5
18	It’s difficult to eat vegetables with meals.	Likert 1-5
19	working out is a hard for me, so I do not do it.	Likert 1-5
20	I feel healthy without engaging in physical activity and only watching TV for a long time.	Likert 1-5
21	There is no benefit to be physically active every day.	Likert 1-5
Attitude on SM		
22	Identifying myself can make me confused.	Likert 1-5
23	Identifying my strengths or weaknesses is not important in my life.	Likert 1-5
24	I do not bother with my good or bad habits.	Likert 1-5
25	Engaging in relaxation activity is a wrong at stressful situations.	Likert 1-5
26	Relaxation activity is difficult to do and is a waste of time.	Likert 1-5
27	Relaxation activity will not benefit me.	Likert 1-5
28	Stress is a harmful disease with no cure.	Likert 1-5
29	Stress can change my life forever.	Likert 1-5
30	Because nobody in my family suffered from stress, I should not worry.	Likert 1-5
31	Good stress management cannot reduce stress.	Likert 1-5
32	Stress management cannot help me to cope with stress.	Likert 1-5
33	Life is full of stress. I cannot manage it.	Likert 1-5
34	Getting advices from a wise person is not suitable to deal with stress.	Likert 1-5
35	Pray to God to reduce stress is not a moral value.	Likert 1-5
36	I do not need to think about moral values in my life.	Likert 1-5
37	Moral value does not affect my relationships with others.	Likert 1-5
Eating Behavior		
1	How frequently do you have breakfast?	Likert 1-6
2	How frequently do you drink morning tea?	Likert 1-6
3	How frequently do you have lunch?	Likert 1-6
4	How frequently do you drink afternoon tea?	Likert 1-6
5	How frequently do you have dinner?	Likert 1-6
6	How frequently do you have dinner?	Likert 1-6
7	How often do you eat out?	Likert 1-6
8	How frequently do you eat at Western fast food restaurants?	Likert 1-6
9	How frequently do you drink carbonated and sweetened beverages?	Likert 1-6
10	How often do you eat sweet foods (cookies, candies, and cakes)?	Likert 1-6
11	How often do you eat fruit?	Likert 1-6
12	How often do you eat vegetables and salad (cooked or raw)?	Likert 1-6
13	How often do you eat/ drink dairy products? (milk, cheese, yogurt)	Likert 1-6
14	How often do you eat fish?	Likert 1-6
Relaxation		
1	How many times did you do muscle relaxation activity in the last week?	Likert 1-5
2	How many times did you do deep breathing activity in the last week?	Likert 1-5

LS, lifestyle; D, diet; PA, physical activity; SM, stress management

* Excluded as a result of CVR test.

**Table 5:** The Result of Convergent Validity of the Reflective Model

**Construct**	**AVE**	**Response ** **Reliability**	**R**	**R** ^2^	**Cronbach’s ** **Alpha**	**Communality**	**Redundancy**
Knowledge on LS, D, PA	0.632	0.712	0.618		0.732	0.698	
Knowledge on SM	0.646	0.796	0.634		0.751	0.674	
Attitude on LS, D, PA	0.614	0.751	0.602		0.783	0.714	
Attitude on SM	0.629	0.715	0.612		0.710	0.693	
DASS	0.532	0.922	0.634	0.494	0.901	0.532	0.0089
Coping	0.705	0.796	0.602		0.819	0.705	
Knowledge	0.614	0.725	0.612		0.711	0.614	
Attitude	0.629	0.740	0.618		0.802	0.629	
GOF =√(AVE × R 2)	0.551	0.796	0.634		0.751	0.674	

**Figure 1:**
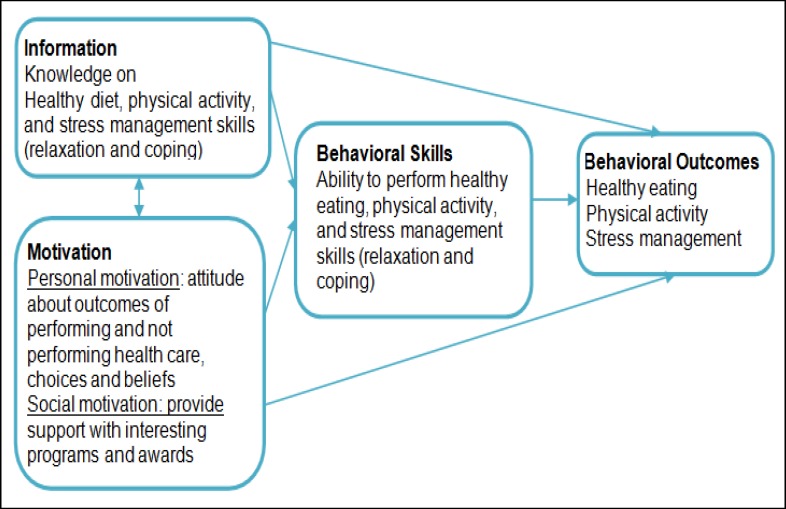
Theoretical Framework for IMB Model (12, 19)

**Figure 2:**
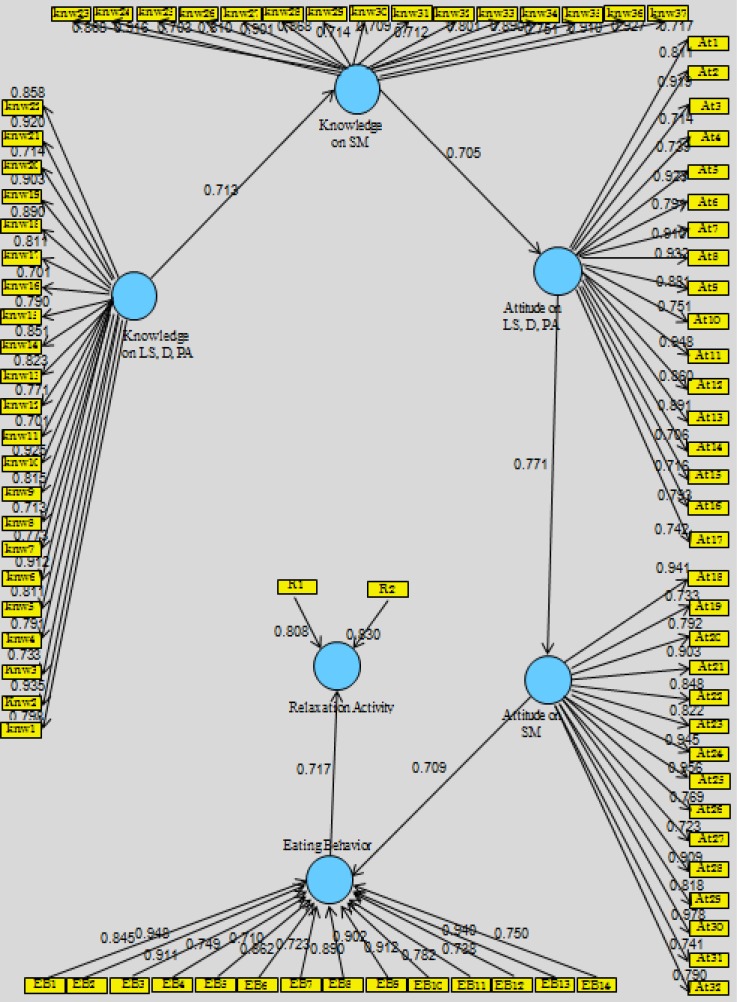
PLS for both Formative and Reflective Researcher-Administered Questionnaires

**Figure 3:**
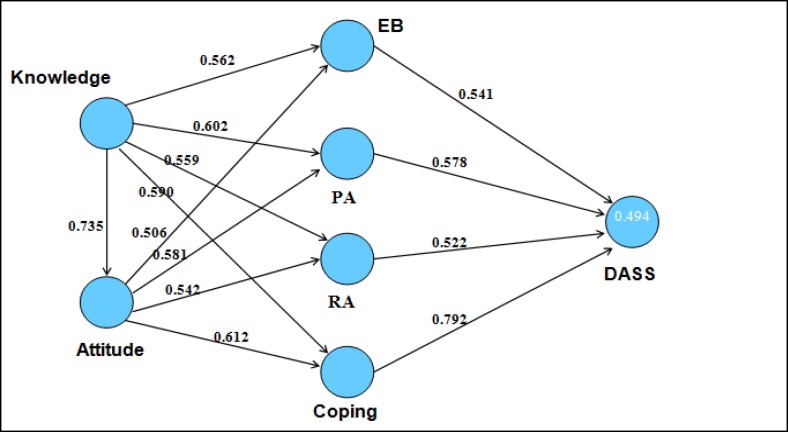
PLS for Theory of IMB Model

## Discussion

In the present study, using the IMB model, the knowledge and attitude of lifestyle, diet, physical activity, relaxation activity, and stress management skills were measured. Variables were entered into the SEM/PLS analysis to adjust the theory and constructs. Similarly, a study by Kelly et al (12) measured knowledge and beliefs to test the IMB model for health promotions among adolescents. They used SEM analysis to set the theoretical constructs.

The PLS (partial least square) is a method with structural advantages when used to conduct the equation models. The PLS specifies the relationships between indicators and latent variables in the same model and can consider measurement errors (63). A study by Rönkkö and Ylitalo valued the validation using PLS (7). They stated that good indicators receive more weight and they are the sign of maximizing construct validity. Moreover, they showed that besides the value of weighting, correlation between associated constructs and the structural paths is important. In addition, they indicated that PLS comprises better measurement in extra correlation of items in different constructs. 

The model contains all the indicators introduced to the SEM analysis. It showed adjusted measurements that significantly differed from the levels considered reasonable, considering the discriminant validity, convergent validity, and reliability. In the initial model, the factorial loads suggested that there was a problem regarding the constructs of the questionnaires and response scales. Factorial loading problems may lead to the incomprehension of the item. Similarly, Maciel et al (51) showed theoretical problems in the initial model, which was corrected after removing problematic constructs. They showed that by removing those items, AVE for other constructs increased.

The main goal of the SEM is to assess the construct validity of items measuring theoretical construct. Items RA and EB showed smaller factorial loads; however, they had acceptable values and caused no problems, so they were able to remain in the construct among the other variables. The SEM model is designed to show the clarity of the relationship between variables, not to depict one individual relationship; it will not even run if implemented on a single variable. In addition, in the interpretation of SEM, the cause-effect relationship does not work, but the predictor relation is accepted (64). Similarly, a study by Hassan Et al tried to show the validation of the e-lifestyle model, as well as the weight and importance of each dimension (65). The study showed that which factors or strategies affect respondents better. They indicated that some factors may seem to be ideal factors, but their weight in the PLS show opposite results. 

 Using SEM requires a theoretical justification to identify the relations proposed (61). The final model declared that the theory of IMB model is responsive for reducing depressive symptoms with related factors. It seems that items, including knowledge, attitude, eating behavior, physical activity, and stress management skills, which are the components of lifestyle, can reduce depressive symptoms. The correlation between DASS and coping was strong, so their correlation was more highlighted. Similarly, Maciel et al (51) stated that product quality was the more highlighted variable because of the strong correlation; however, knowledge and fish nutritional value are not very important. 

In this study, in the initial model, only 11 variables were removed, but there was no change in the overall framework and theory. On the contrary, in some studies, the final model and the initial model were not consistent with each other and the constructs were changed a lot (51). According to the literature review for IMB model, knowledge and attitude are the main aspects for the adjustment. Therefore, the path analysis starts with knowledge and attitude and moves on to practices such as diet and physical activities. Maruayama (55) investigated human behavior as a format of consumer behaviors and showed that perfect reliability is unreal. 

These study findings demonstrate the prominence of the IMB model for predicting healthy lifestyle and reducing depressive symptoms in adolescents. The fit of the data was suitable for the whole sample. In the measurement portion of the model, there were adequate indicators for each latent variable.

## Limitation

This sample was taken from high school students in Kuala Lumpur and does not represent adolescents in the whole country. It is better to extend it to a wider population across the country or other societies. Some of the measures, including physical activity, coping, and DASS-21, were validated and standard questionnaires, and some were researcher-administered questionnaires, which were validated through this study.

## Conclusion

The IMB model, with theoretically based constructs, was correlated with healthy lifestyle components to predict depressive symptoms among adolescents using the PLS. PLS considers that there were good enough correlations between different items of the construct. Thus, the weights of indicators appeared to be appropriate indicators for the model. The proposed model was based on the literature review (12). The use of SMART-PLS software ensured the validity and reliability of the model; however, some problematic variables were removed to increase the validity of the model. Developing methods to assess the lifestyle components may help establish a healthy lifestyle among adolescents. The results of this study proved that the information, motivation, behavioral skills model is efficient for healthy lifestyle and can be a good base for further research. Further theory may allow a wider range of variables such as community influences and self-efficacy. To improve the validity of the model, further validation through a larger population is recommended using different structural models.
